# Role of Nutrition in Pediatric Patients with Cancer

**DOI:** 10.3390/nu15030710

**Published:** 2023-01-30

**Authors:** Laura Pedretti, Serena Massa, Davide Leardini, Edoardo Muratore, Sofia Rahman, Andrea Pession, Susanna Esposito, Riccardo Masetti

**Affiliations:** 1 Pediatric Clinic, University Hospital, Department of Medicine and Surgery, University of Parma, 43126 Parma, Italy; 2 Pediatric Unit, IRCCS Azienda Ospedaliero-Universitaria di Bologna, 40138 Bologna, Italy

**Keywords:** cancer, enteral nutrition, gut microbiome, malnutrition, nutrition, parenteral nutrition

## Abstract

Children with cancer are at high risk for developing short-term and long-term nutritional problems related to their underlying disease and side effects of multimodal treatments. Nutritional status (NS) can influence several clinical outcomes, such as overall survival (OS) and event-free survival (EFS), treatment tolerance, risk of developing infections and quality of life (QoL). However, the importance of nutrition in children with cancer is still underestimated. This review focuses on alterations of NS that occurs in children during cancer treatment. In particular, we reviewed the pathogenesis of undernutrition in oncological children, as well as how NS affects treatment tolerance and response, the immune system and the risk of infections of children with cancer. Thanks to recent advances in all types of supportive therapy and to the progress of knowledge on this topic, it has been realized that NS is a modifiable prognostic factor that can be intervened upon to improve the outcome of these patients. Currently, there is a lack of a systematic approach and standard recommendations for nutritional care in the pediatric cancer population. Literature analysis showed that it is essential to define the NS and treat any alterations in a timely manner ensuring proper growth and development. Nutritional follow-up should become an integral part of the care pathway. Regular nutritional monitoring should be performed at diagnosis, during treatment and during follow-up. A close collaboration and sharing of expertise between pediatric oncologists and nutrition specialists, combined with careful and participatory sharing of the feeding experience with the family and the child (after age 6 years), is strongly required.

## 1. Introduction

Undernutrition is a condition in which there is inadequate nutritional status (NS) [1]. It is the result of an imbalance between the energy and nutrients supplied and those the body needs. In children, it is defined by the World Health Organization (WHO) as both undernutrition and overnutrition, as reported in Table 1 [2]. Undernutrition is classically subdivided into acute undernutrition or wasting, defined by the WHO as weight-for-height (WFH) < −2 standard deviations (SD), and chronic undernutrition or stunting, defined by the WHO as height-for-age (HFA) < −2 SD. For children, age needs to be considered when defining overweight and obesity. For children aged 5–19 years, overweight is defined as BMI (body mass index)-for-age > +1 SD and obesity is defined as BMI-for-age > +2 SD. For children under 5 years of age, overweight is defined as WFH > +2 SD and obesity is defined as WFH > +3 SD [3,4].

Advancements in supportive care areas such as pain management, emesis control and infection prevention and treatment have contributed not only to improved survival now exceeding 80% in high-income countries but also to a better quality of life for children undergoing cancer treatment [5,6]. Children with cancer are at high risk of nutritional deficits due to cancer itself, the toxicity of therapies and their increased physiological requirements. However, the importance of nutrition in children with cancer is still underestimated. The incidence of undernutrition among children and adolescents undergoing cancer treatment is reported to be 0–70%, whereas the incidence of overnutrition is reported to be 25–75% [7]. The variability of incidence data is probably due to many confounding factors, such as the lack of a unique shared definition of undernutrition, the different types of disease and stages, the intensity and length of therapy, and the small sizes of studies conducted [8]. Studies on this topic focus mainly on hematological diseases and on the aspect of undernutrition during cancer treatment, with overnutrition being overlooked. 

NS during cancer therapies affects several clinical outcomes, such as overall survival (OS) and quality of life (QoL). NS influences the risk of morbidity and mortality both during and beyond the treatment of cancer, impacting the long-term health of survivors [9,10]. Undernutrition has been identified as a relevant risk factor for the development of infections. Undernutrition has been seen to increase infectious risk and to influence survival [11]. In addition, hyperglycemia, a complication of the therapies used in the treatment of pediatric cancer patients, has been associated with an increased risk of infections and reduced survival in pediatric cancer patients [12,13,14]. Long-term health outcomes are influenced by late-treatment-related effects, such as endocrinopathies, reduced bone mass, metabolic syndrome, cardiovascular disease, obesity, and hypertension [15]. Indeed, children are at a growth and development stage and require additional energy. Undernutrition problems, during this critical phase, may compromise proper growth, puberty and the correct development of the child, and balanced body composition [10]. 

The gut microbiome (GM) has been at the center of attention during recent years as a contributor to the origin and progression of some diseases. The microbiome seems to play an important role within oncological pathophysiology. Chemotherapy causes gastrointestinal (GI) toxicity, with changes in the normal bacterial flora, determining dysbiosis and contributing to the patient’s undernutrition [16]. Some foods, such as ready-to-use therapeutic foods (RUTFs), and some probiotics, such as Bifidobacterium, could modulate microbiomes, restoring eubiosis and providing adequate nutrition and even modulating the efficacy of cancer treatment [14,17]. 

For all the reasons mentioned above, nutritional follow-up should become an integral part of the care pathway. Currently, there is a lack of a systematic approach and standard recommendations for nutritional care in the pediatric cancer population. This review focuses on alterations of NS that occur in children during cancer treatment. Specifically, we reviewed the pathogenesis of undernutrition in children with cancer and how NS affects treatment tolerance and response, the immune system, and the risk of infections. 

## 2. Pathogenesis of Malnutrition in Childhood Cancer 

Undernutrition in pediatric cancer patients is caused by a combination of factors, including the underlying disease, host inflammatory response, side-effects of cancer therapy, and increased metabolic demands. These factors lead to changes in dietary intake, physical activity levels, and ratio of lean to fat mass, resulting in an alteration of the energy balance [7]. In cancer patients, there is a microenvironment of proinflammatory cytokines (TNF-α, IL-1, IL-6, IFN-γ) released by the tumor that causes accelerated mobilization and oxidation of energy substrates, increased lipolysis, and loss of whole-body proteins. Cytokines may also act directly on the central nervous system (CNS), affecting appetite and increasing energy expenditure [18,19].

Moreover, each type of cancer therapy, such as surgery, radiation therapy (RT), and chemotherapy (CHT), can produce several specific side effects. The combination of these therapies, which occurs in most therapeutic protocols, affects the NS by decreasing appetite and oral intake or inducing nausea and vomiting [17]. This can lead to loss of fluids, alteration of electrolytes, and deficiency of proteins and macro- and micro-nutrients [16]. Cytotoxic effects of CHT and RT can cause mucositis, which can affect the mucosa of the oral cavity and GI tract. Lesions of oral mucositis are often very painful and compromise oral intake and oral hygiene, increasing the risk of local and systemic infections [7,20,21]. 

Many chemotherapy drugs used to treat leukemia have been found to cause oxidative stress in vitro [22]. However, the connection between nutrition and oxidative stress remains under investigation. Cellular oxidative stress is associated with both dietary intake and cancer therapy. Oxidative stress, which can cause disease progression and stress-induced DNA damage under normal conditions, is believed to enhance the effectiveness of certain chemotherapy regimens in treating pediatric leukemia and lymphoma patients. The relationship between certain dietary compounds and the level of oxidative stress during chemotherapy may provide some molecular basis for how nutrition affects patient outcomes during treatment. Raber et al. investigated the correlation between the nutritional status of pediatric cancer patients and changes in direct measures of oxidative stress [22]. This study specifically looked at pediatric cancer patients undergoing chemotherapy for leukemia and lymphoma and monitored their nutritional status and changes in oxidative stress over a six-month period. The results showed that oxidative stress levels increased over time during the period of follow-up and were linked to protein consumption, including both animal and vegetable protein [22].

There is a major controversy over whether the metabolic rate of children with cancer is different from that of children of the same age and sex without cancer [23,24]. A study by Broeder et al. found that the basal metabolic rate (BMR) of children with solid tumors increased at diagnosis, decreased during the therapy, and normalized at the end of therapy, noting that the tumor itself was the cause of the patients’ increased metabolism. Therefore, the tumor is an active metabolic tissue that increases basal energy demands [23]. Other authors found that the BMR of children with cancer is not significantly different from healthy controls and that there is no evidence of raised energy expenditure. Furthermore, cancer patients present abnormalities in energy metabolism but are not in a hypermetabolic condition and data do not support the hypothesis that energy expenditure is uniformly elevated in cancer patients [25,26]. 

In cancer patients, depletion of skeletal muscle happens due to an altered balance between synthesis and degradation of proteins and increased myocyte apoptosis [7,8]. Loss of lean body mass can occur rapidly in pediatric cancer patients, leading to significant alterations, such as reduced muscle strength, weakened immune function, delayed wound healing, and increased morbidity [7,8]. This loss of lean mass may be accompanied by an increase in body fat stores, as seen with large and prolonged doses of glucocorticoid (GCS) therapy. GCS can promote central fat accumulation, reduce adipocyte insulin sensitivity, and inhibit growth hormone (GH) responses, resulting in an enhancement of the somatostatin effect at the pituitary level [7,8,19].

In pediatric cancer patients, there are additional energy requirements compared to adults, as children are in a phase of growth and development [15]. If these requirements are not met, the linear growth pattern may be compromised. Linear growth can be adversely affected by endocrine complications due to the disease itself as well as by the treatments used. GH deficiency (GHD) is the most common pituitary disorder in pediatric cancer patients, and it is more pronounced after craniospinal RT for brain tumors and total body RT for bone marrow transplantation, resulting in linear growth failure [27]. Cranial irradiation may also contribute to the development of obesity and metabolic syndrome due to hypothalamic–pituitary axis damage. In particular, patients with brain tumors have a high risk of hypothalamic–pituitary axis damage due to cancer therapies and the location of the tumor, leading to abnormalities in the satiety and hunger control mechanism and, as a consequence, an increase in the prevalence of obesity during and after treatment [19,28]. 

**Figure 1:**
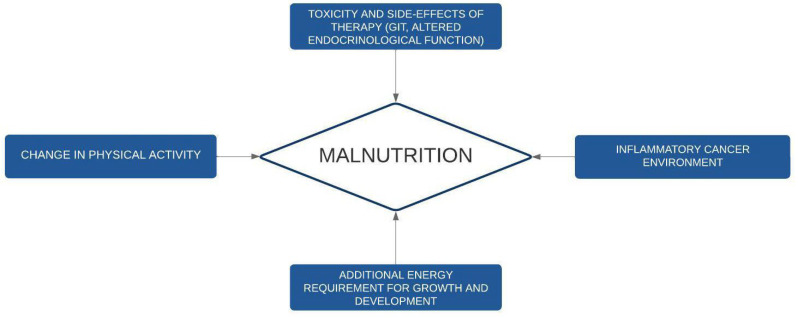
Pathogenesis of undernutrition in childhood cancer. GIT: gastrointestinal toxicities.

In Figure 1, we summarize the pathogenesis of undernutrition in cancer pediatric patients.

## 3. Methods to Analyze Nutritional Status in Children with Cancer

It is important to assess NS at diagnosis and to monitor NS during treatment as well as during survivorship. This is because, if the initial assessment is not carried out promptly at diagnosis, there is a risk of having results altered by the procedures and therapies used [17]. The aim is to ensure proper NS and regular development [16]. 

Nutritional assessment should consider the NS of the patient, GI function, intensity of treatment and current or expected side effects of treatment. A recent consensus statement, performed by Fabozzi et al., proposed a timing of nutritional assessment during pediatric cancer treatment [29]. Children receiving intensive treatment or at high risk of malnutrition should be evaluated at a maximum interval of 3–4 weeks. Children on less intensive treatment should be optimally evaluated at an interval of 3 months and at 6 to 12 monthly intervals during the maintenance phase of treatment. More frequent reassessments are needed in children admitted to intensive care units (ICU) (Table 2).

Nutritional assessment and guidance should be extended through survivorship. NS should be periodically evaluated to avoid overlooking any changes in survivors and to identify patients who need advanced nutritional support [30]. Viani et al. proposed a plan for nutritional assessment for childhood cancer survivors [31]. Patients without nutritional risks can be evaluated every 6 months during the first year of follow-up and yearly after the first year. Well-nourished patients with nutritional risk factors (i.e., inadequate eating habits, sedentary lifestyle, hypertriglyceridemia, high cholesterol levels) should be evaluated every 3 months during the first year, every 6 months until the fifth year and then annually. Undernourished children require monthly assessment until they achieve normal NS. Obese children need revaluation every 3 months [29,31]. Table 3 summarizes the proposed timing of nutritional assessment into survivorship.

The A-B-C-D method is a standardized tool recommended for NS screening in children with cancer [29,32]. It includes anthropometric measures (A), biochemistry exams (B), clinical evaluation (C), and dietary intake (D) [32]. The evaluation of NS is based on the use of the anthropometric parameters. Traditional anthropometric measurements, such as BMI, are considered inadequate indicators of NS in children with cancer [7,19]. In these patients, fluid balance may be altered, especially in the presence of edema due to treatment and in the presence of an abdominal tumor mass, such as neuroblastoma, Wilms tumor, or hepatoblastoma [16]. Under such conditions, there is a risk of underestimating undernutrition, especially when considering only BMI, which is unable to distinguish between fat mass and lean mass. This distinction is important, as cancer patients may experience a selective loss of lean mass in favor of an increase in fat mass, despite a stable body weight [10].

The use of arm anthropometry allows for more sensitive data on NS in these cases than BMI. Triceps skinfold thickness (TSFT) is an indicator of fat mass, while middle-upper arm circumference (MUAC) is a measure of lean body mass [8]. In children under 3 years of age, it is also important to assess head circumference, which is fundamental for proper neurodevelopmental outcome [16], but we must consider that it may be impaired in patients with brain tumors. The measurement of lean body mass is important to accurately assess NS as it represents the most metabolically active component of the body. Different systems can assess body composition, distinguishing muscle mass from fat mass. Bioelectrical impedance (BIA) allows rapid estimation of fat and lean tissue mass using impedance of electrical current and avoiding exposure to radiation [8]. Accuracy is reduced in the case of oscillating hydration status and in chronically ill patients. Imaging with dual-energy X-ray absorptiometry (DXA) differentiates lean and fat tissues though specific pediatric equations, but it does not distinguish visceral from subcutaneous fat [8,33]. Thanks to advances in imaging techniques, computerized tomography (CT) and magnetic resonance imaging (MRI) can now distinguish visceral and subcutaneous adipose tissues, as well as skeletal muscle mass, from other lean tissues [33].

Muscle health and function are impaired in both pediatric cancer patients and survivors [34]. The musculoskeletal effects such as loss of mass and strength that can be seen in cancer patients are due to CHT and RT used at a crucial period of physiological development. Hand-held dynamometry (HHD) is a cost- and time-efficient method that can provide a basic quantifiable measure of strength [34].

Anthropometric and body composition data should be supplemented with laboratory data including liver and renal function tests, a lipid and glucose panel and serum protein concentrations, such as albumin, pre-albumin, retinol-binding protein (RBP) and transferrin [35]. These proteins are most influenced by fever and infection/inflammation, fluid shifts, liver and renal dysfunction, and by the use of drugs such as asparaginase or GCS. Albumin has a long half-life (3–4 weeks) and is more suitable for defining chronic undernutrition [35]. Its value can be affected by protein-losing enteropathy, renal losses, or burns. Liver toxicity due to cancer therapy can also reduce the rate of albumin synthesis. Albumin serum level is also conditioned by its supplements, which cancer patients may be subjected to during oncological treatment [35]. Prealbumin has a shorter half-life (2–3 days), which makes it a better indicator of acute state changes in NS rather than overall nutritional status [7,8]. Thanks to its rapid turnover, prealbumin may more identify the effectiveness of nutrition therapy [36]. RBP represents the visceral protein with the shortest half-life (approx. 12 h). Similar to prealbumin, RBP is a tool for evaluating short-term effects of nutritional modifications because of its rapid turnover [37]. However, RBP is more difficult to measure than prealbumin [37,38]. Both pre-albumin and RBP levels can be affected by vitamin A deficiency. Transferrin, with a half-life of 8–9 days, is a predictor of death in children with protein energy undernutrition, and its levels increase during infections and iron deficiency [35]. A comprehensive nutritional analysis should assess dietary intakes of micronutrients such as B vitamins, vitamin D, calcium, and zinc, as deficiencies in these substances can lead to increased toxicity of therapy and more morbidities [7]. Most of these tools are currently under study, and clinical applicability is yet to be evaluated.

Clinical evaluation is essential to detect signs of undernutrition. Assessing subcutaneous fat loss or excess, muscle wasting, skin and hair changes, recent weight variation, presence of edema, mucous membrane dryness, and evidence of vitamin and mineral deficiencies is crucial in children with undernutrition [31]. It is also important to evaluate the side-effects of cancer treatment on oral food intake, such as the presence of vomiting, loss of appetite, diarrhea, constipation, flatulence, belching or indigestion, mucositis, nausea, dysphagia, taste aversions, xerostomia, and inability to chew and swallow [31].

An adequate nutritional history should be taken at the initial assessment and re-evaluated thereafter to reveal typical side-effects affecting dietary intake [30,36]. The intake of macro- and micronutrients, food aversions, allergies or intolerances, current eating patterns, changes in physical activity level, family behavior, and food hygiene at home should be investigated. A food diary recording all intake for 3 to 7 days is a useful tool in this process [30,36]. This evaluation should be performed by expert personnel, such as dietitians or clinical nutritionists with expertise in this area [29]. Thanks to advances in technology, numerous digital tools can be used to facilitate dietary intake monitoring.

Various nutritional screening tools have been developed to assess child’s undernutritional risk [31]. However, there is not enough evidence to choose one over another. Strong Kids is an easy-to-use screening tool for hospitalized children based on four items: subjective clinical assessment, high-risk disease, nutritional intake and losses (diarrhea, nausea, vomiting), and weight loss or poor weight gain [18]. This tool considers cancer as a single entity without differentiating the types of tumors and the stage of treatment [29,39]. The Patient-Generated Subjective Global Assessment (PG-SGA) is a simple, sensitive tool that is capable of identifying nutritional risk in newly diagnosed pediatric cancer patients [40]. It is non-invasive and can be conducted by a health care professional at the bedside yielding immediate results. This score allows malnourished hospital patients to be identified and triaged for nutritional support. The score consists of information about history of weight loss, nutritional symptoms, changes in dietary intake during the last month, functional capacity, and physical exploration, as well as the presence of ascites and edema [40].

It would be useful to develop a disease-specific nutrition score that also takes into account the disease and intensity of treatment. Therefore, it is necessary to integrate anthropometric parameters, laboratory data, body composition tests, caloric intake assessment, and energy expenditure to better define the NS of children with cancer. In Table 4, the various steps of the nutritional assessment are summarized.

## 4. Prevalence of Undernutrition in Different Cancer Types

Available studies on the prevalence of undernutrition (both overnutrition and undernutrition) in pediatric cancer patients are characterized by considerable heterogeneity. The prevalence varies based on the type of tumor (its localization/staging and its biological behavior), the type of intervention, the age of the patients, and the methods and cut-off points used for the assessment of NS [1,16]. In general, undernutrition is prevalent at diagnosis and worsens during the therapy due to the cancer itself and the chemotherapy, while overnutrition is more prevalent at the end of treatment, but it can also be present at the start of treatment, especially in patients with brain tumors or those receiving high doses of steroids [1,16].

A lower prevalence of undernutrition was found in patients with acute lymphoblastic leukemia (ALL), lymphomas, and non-metastatic local tumors, and patients in remission undergoing maintenance chemotherapy [41,42]. In general, according to the literature, the prevalence of undernutrition in leukemia is 5–10% at diagnosis and up to 5% during treatment, 50% in patients with neuroblastoma at diagnosis and 20–50% in neuroblastoma during treatment, and up to 30% in other malignant tumors at diagnosis and during treatment. It is challenging to estimate the prevalence of undernutrition in patients with solid tumors due to the lack of appropriate studies, and even fewer studies have been done on brain tumors [41].

Two retrospective studies were conducted on leukemia patients [43,44]. The first study found that 7.6% of male and 6.7% of female patients were undernourished at diagnosis, while 2.6% of males and 3.2% of females were overnourished in ALL patients [43]. This study also suggested initial screening for nutrition to quickly recognize and address undernutrition, using calculation of the BMI [43]. The second study reported a 10.9% prevalence of underweight and 14.9% prevalence of overweight in acute myeloid leukemia (AML) patients [44]. These patients, with BMI below the 11th percentile, had a lower chance of survival than normal-weight children, possibly due to their decreased ability to fight infections [44].

As stated before, studies of solid and brain tumors have limited data, with one study reporting a 50% prevalence of undernutrition at diagnosis for solid tumors, decreasing to 33% after two courses of CHT and 20% after excision of the tumor. Children with solid tumors were found to be at high metabolic risk [44]. A study has shown that pediatric patients with neurological tumors had an undernutrition rate of 31% at diagnosis [45]. Undernutrition can be even greater in children diagnosed with medulloblastoma, who present recurrent symptoms of emesis and nausea [46]. Brain tumor patients had the highest rate of overnutrition at diagnosis, compared to other tumor groups, with rates of 42.6% and 40.4% for overnutrition and obesity respectively [1].

Table 5 summarizes the prevalence of undernutrition in different cancer types.

## 5. Consequences of Undernutrition in Children with Cancer

Undernutrition during treatments can negatively impact the tolerance of CHT, OS, EFS, risk of infection, and QoL [7].

Indeed, undernutrition can negatively impact cancer patients’ QoL, both directly and indirectly. It can lead to a decrease in bone mineral density (BMD) and an increase in the risk of chronic diseases such as cardiovascular diseases, diabetes, and metabolic syndrome. Additionally, undernutrition can make patients more susceptible to infections and alter drug metabolism, leading to increased toxicity. These factors can lead to delays in treatment, poorer overall survival and event-free survival, and an increased risk of relapses [7,15,16].

**Figure 2:**
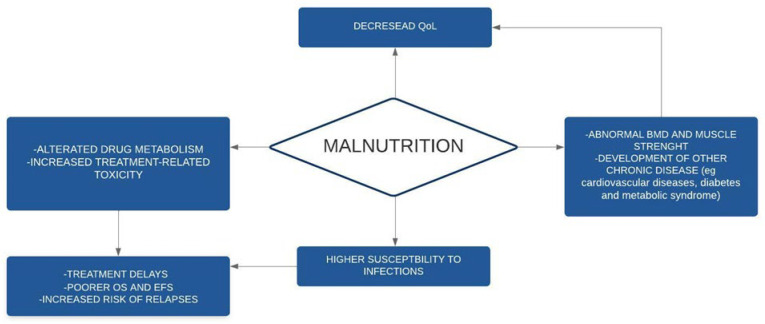
Consequences of malnutrition in children with cancer. BMD: bone mineral density; EFS: event-free-survival; OS: overall survival; QoL: quality of life.

A summary of the main consequences of undernutrition is given in Figure 2.

### 5.1. Effect of Nutrition on Drug Pharmacokinetics

Poor nutrition has been linked to increased treatment-related toxicity [8]. Reduced weight and changes in body composition can have pharmacokinetic implications by affecting CHT volume of distribution, absorption, metabolism, and clearance from blood, resulting in reduced tolerance to treatment. Protein-caloric undernutrition can also alter the renal and hepatic metabolism of certain chemotherapeutic agents, leading to an increase in adverse effects, such as profound myelosuppression and febrile neutropenia (FN) [7,15]. In obese patients, changes in body composition and organ function may be associated with altered serum protein binding, metabolism, and clearance of chemotherapeutic drugs. For hydrophilic drugs, the excess of fat mass is not available for distribution, resulting in a lower total volume of distribution, while lipophilic drugs have a higher volume of distribution [15,16]. Adiposity is also implicated in the production of tumor growth factors, such as insulin-like growth factor 1 (IGF-1), and in resistance to the cytotoxic effects of CHT due to a protective effect on tumoral cells [10]. The increased toxicity of the therapies, in association with the increased risk of infection due to immune incompetence, can lead to treatment delays and cessation, which can result in increased relapses and inferior survival [10].

### 5.2. Impact on Survival Rate

Children with ALL or AML with a higher BMI at diagnosis have poorer survival [47]. Poorer EFS has been observed in ALL children with a higher BMI (above 85 percentile), with significantly increased mortality and a statistically nonsignificantly greater risk of relapse [47]. Patients with ALL who are obese at the induction of therapy also have a significantly increased risk of persistent minimal residual disease (MRD) in the bone marrow [48]. The impact of weight on EFS does not occur exclusively at diagnosis. Persistence of high weight during treatment also has a negative impact on the risk of recurrence and death [49]. Obese or underweight patients with ALL who maintain their altered NS for more than half of the duration of the pre-maintenance therapy have a double risk of future relapses or death compared to those who remain normoweight or those who reach normoweight during treatment [49]. In these two groups of patients (obese and underweighted), the EFS is inferior (hazard ratios, 1.43 and 2.30, respectively; global *p* < 0.001) [49]. Normalizing weight during therapy reduces this risk, making it comparable to never being obese or underweight. This suggests that nutritional interventions may modify outcomes in cancer patients [47,50].

Poorer EFS and OS and increased treatment-related mortality have been recorded in AML children with a higher BMI. More frequent abdominal pain, hypertension, and pulmonary and coagulation problems have been found in overweight children with AML [44,47].

Joffe et al., in a recent systematic review, studied the influence of BMI on both survival and treatment toxicity in a heterogenous population of pediatric patients with solid tumors arising from renal, bone, liver, eye, muscle, vascular, germ cell, and neural crest tissues. Abnormal BMI was associated with worse OS in Ewing sarcoma, osteosarcoma, and a trend towards poorer OS in rhabdomyosarcoma. High BMI in osteosarcoma was associated with increased nephrotoxicity and post-operative complications [51].

Undernutrition is associated with poorer survival and with decreased survival rates in both hematological and solid tumors. Loeffen et al. demonstrated, in a heterogeneous childhood cancer population (269 patients, including 139 with hematological malignancy, 86 with solid tumors and 44 with brain tumors), that survival was significantly worse at diagnosis or 3 months after diagnosis for patients who were undernourished than for those who were well- and over-nourished at diagnosis [52]. This topic is significant because, unlike undernutrition at diagnosis, undernutrition 3 months after diagnosis is possibly preventable by close follow-up of the NS and rapid intervention if necessary, and consequently could improve survival [52].

In leukemia patients, undernutrition plays a negative prognostic role, especially in low-middle-income countries. Barr et al. showed that undernutrition was associated with a larger number of dropouts from therapy and a lower 2-year EFS [53]. In addition, it was demonstrated that undernourished children with leukemia had much poorer 5-year disease-free survival and presented more illness compared to well-nourished children at the same age. The early identification of an underweight patient with leukemia should be the first step to act on long-term survival [53].

In patients with advanced neuroblastoma, Wilms tumor and Ewing sarcoma, the prevalence of undernutrition is particularly high (average 34%) [33]. Weight loss at diagnosis is a risk factor for the development of suboptimal NS during cancer treatment, and NS at the time of diagnosis can affect outcomes in terms of morbidity and mortality [1]. Patients with undernutrition in the initial phase of therapy have worse OS, independent of expected survival based on diagnosis. Undernutrition in patients with solid tumors was more frequent from diagnosis until the end of therapy [54]. The risk of undernutrition is high, especially in the first 3 months of treatment, particularly in high-treatment-risk patients. Undernourished patients at diagnosis were statistically significantly associated with a high risk of relapse, death or becoming palliative, and were 14 times more likely to have one of these events [55].

### 5.3. Undernutrition and Infections

Infections are a common cause of morbidity and mortality in pediatric cancer patients [30]. The pathogenesis of infectious disease in pediatric patients with cancer is multifactorial. The risk of infection can be divided into disease-associated factors (such as bone marrow suppression typical of hematological disease and the resulting risk of developing infections), treatment-related factors (such as CHT-induced neutropenia) and patient-related factors (such as age, comorbidities, and NS). Among patient-related factors, NS is considered a relevant risk factor for developing infections. Undernutrition increases the risk of severe infection complications. The catabolic state and nutrient depletion typical of cancer diseases increase the risk of sepsis in pediatric cancer patients [7].

Undernutrition reduces immune defenses and increases the risk of infections and FN in pediatric cancer patients by causing hormonal changes and a compromised cytokine response [18,53]. Nutritional deficiencies affect B and T lymphocytes, polymorphonuclear cells (PMNs), mononuclear phagocytes, complement system function, and cytokine immunoregulation [30].

A low BMI at baseline and significant weight loss during treatment courses are strong prognostic indicators linked with both bacterial and fungal infections and lower survival [11]. A study by Loeffen et al., conducted on a group of pediatric cancer patients, showed increased rates of FN episodes with bacteremia in the first year after diagnosis in children who lost more than 5% of their body weight in the first 3 months after diagnosis [52]. Therefore, rapid weight loss can make patients more vulnerable to bacterial infections. On the other hand, weight loss between 3 and 6 months and between 6 and 12 months after diagnosis did not affect the occurrence of FN episodes [52]. The same result regarding the strong association between rapid weight loss in the first 3 months after diagnosis and increased FN was found in a retrospective study performed by Triarico et al. [18]. In this study, a weight loss ≥5% in the first 3–6 months after diagnosis strongly increased the occurrence of ≥3 hospitalizations for FN. This study showed that malnutrition at diagnosis is an independent factor that significantly increases hospitalization for FN [18]. Overall, the results of these studies may aid in identifying patients who are at risk of malnutrition and, as a result, at risk of developing infection complications [18,52].

Hyperglycemia is a known complication of the therapies used in the treatment of pediatric cancer patients. GCS and asparaginase are the most frequent agents responsible for hyperglycemia [12,13,14]. The prevalence range of hyperglycemia during cancer treatment is 10% to 20%, and it is greatest during the treatment of ALL [12]. Hyperglycemia is a complication of induction CHT in 10% to 50% of pediatric patients with ALL. Hyperglycemia has been associated with an increased risk of infections and reduced survival in pediatric cancer patients [12,13,14]. It is not entirely clear whether hyperglycemia is a clinical marker for infection or has direct biological causality. However, numerous studies have shown how acute hyperglycemia affects innate immunity and the ability to fight infections [13].

Currently, there are no set guidelines for monitoring hyperglycemia during pediatric cancer therapy, in general or among specific disease groups. There are no standard indications for how often, which measurements, or how or when to intervene. However, pediatric patients undergoing cancer treatment should be evaluated with serial blood glucose assessments for potential risk of hyperglycemia, particularly patients aged over 10 years and those with Down syndrome, CNS involvement, obesity, or planned therapy that includes glycemic-altering agents such as GCS and asparaginase [12].

### 5.4. Impact on the Psychological Sphere and QoL and Other Long-Term Consequences of Malnutrition

Malnutrition can have negative effects on the psychological well-being and QoL of children with cancer [1]. It can specifically affect the emotional and cognitive sphere, social interaction, and physical functioning.

Cancer-related fatigue is a significant problem that affects children undergoing cancer treatment and can persist even after treatment ends [56]. It is characterized by feelings of tiredness, lack of energy, and difficulty engaging in daily activities. Poor sleep quality and decreased physical performance have also been linked to cancer-related fatigue. Additionally, it can lead to depression and negatively impact cognitive function. To improve QoL in children with cancer, it is important to develop non-pharmacological interventions to reduce fatigue. Examples include exercise, massage, music therapy, and health education. One potential intervention is the use of beetroot juice as a supplement during cancer treatment. Beetroot and its constituents have the potential to counter exercise intolerance and fatigue in athletes and diseased populations. However, its effectiveness in children with cancer is not yet clear and needs to be further studied to evaluate the safety and potential efficacy of beetroot juice supplementation to reduce fatigue during exercise and to determine the potential synergistic effects with standard cancer drugs [56].

Long-term consequences of toxic cancer therapies and consequent undernutrition represents an issue to be considered among pediatric cancer survivors. Dyslipidemia, overweight/obesity, and metabolic syndrome are common disorders in surviving patients, and care providers are increasingly having to deal with these problems [57,58]. The sequelae of dyslipidemia have health-related physical outcomes such as high blood pressure, type 2 diabetes, metabolic syndrome, fatty liver disease, orthopedic problems, sleep apnea, and psychological or social problems. Moreover, thyroid disfunction has an impact on metabolic disorders. Hypothyroidism is the most frequently encountered thyroid disorder, and it can be either in primary form, in the case of direct irradiation to the neck, or in secondary form, after craniospinal irradiation. A study conducted by Barg et al. evaluated the prevalence of long-term metabolic disorders in pediatric patients with solid tumors [58]. Lipid disorders, overweight/obesity and thyroid dysfunctions were analyzed in patients who had received their last anti-cancer therapy after at least 1 year (some patients were above 5 years). The results of the study showed an increased risk for the development of dyslipidemia and consequently atherosclerosis in surviving patients. Lipid disorders were found in both proper-weight and overweight patients. Hypothyroidism was diagnosed in surviving patients, but none of the patients with CNS involvement showed thyroid disorders [59]. A study by Bis et al. investigated changes in plasma lipid parameters, thyroid hormone disorders, and the time of their onset in pediatric patients after HSCT [59]. Most of the lipid disorders appeared within six months after HSCT, with the most common being increased triglycerides, decreased HDL level, and elevated total cholesterol level. A downward trend in lipid disorders was observed 6 months after transplantation, although an increase in the occurrence of lipid disorders, particularly LDL levels, was observed one and a half years after transplantation. Similarly, most of the thyroid hormone complications and increased levels of anti-thyroid peroxidase antibodies were revealed within six months after HSCT. Thyroid hormone abnormalities were evenly distributed in time until 4 years after transplantation, with no new occurrences later. Anti-thyroid peroxidase tended to decrease over time but was still observed four to six years after HSCT. Anti-thyroglobulin antibodies showed a rising trend up to one and a half years after HSCT and, after a period of stable detection, peaked 4–4.5 years after transplantation. Due to the frequent occurrence of lipid disorders within six months after HSCT, pediatric patients should follow a diet that restricts saturated fat and includes foods rich in unsaturated fatty acids. Limiting saturated fatty intake and increasing polyunsaturated fat intake can help reduce triglyceride and LDL levels [59].

All the findings reported above highlight that metabolic status, with a focus on lipid profiles, should be closely monitored in pediatric cancer survivors, as they are at higher risk for dyslipidemia and atherosclerosis than healthy individuals [58]. Additionally, thyroid function should be closely monitored, especially in patients who had received radiation therapy and pediatric transplant recipients, in whom thyroid function and associated antibodies must be assessed at least 4 years after HSCT [58,59].

Cardiovascular disease is one of the primary non-malignant causes of morbidity and mortality in childhood cancer survivors [60]. It can be caused by cancer treatments, such as cardiotoxic chemotherapy with anthracyclines and chest irradiation, and can occur prematurely in survivors. In addition to treatment, hypertension, obesity, diabetes mellitus, smoking, and dyslipidemia are relevant risk factors for the development of cardiovascular disease, just as they are in the general population. These are modifiable risk factors, and strategies should be implemented to reduce their incidence among surviving patients. Reducing sodium intake in childhood cancer survivors, especially those with hypertension, could lower blood pressure and possibly reduce cardiovascular events and mortality [60].

Furthermore, to reduce the incidence of obesity and related complications, Zhang et al. proposed an early lifestyle intervention for obesity prevention in pediatric survivors of ALL aged 3–11 years during maintenance treatment or within two years post-treatment [61]. The intervention consisted of a 12-week remote program delivered through web-based sessions and phone calls with a lifestyle coach. The study did not show significant changes in physical activity or weight status, but parents reported reducing the “pressure to eat” feeding practice. Further studies are needed to evaluate the long-term effectiveness of this intervention for pediatric ALL patients and other pediatric cancer patients and survivors [61].

Insufficient intake of bone-related nutrients, such as vitamin D and calcium, which is typical of undernutrition, increases the risk of osteopenia, which is one of the late adverse effects of oncological treatment [7]. The factors underlying alterations in bone metabolism in cancer patients are multiple and act in synergy, including the direct impact of the disease (e.g., leukemia) on the skeleton and the toxic effects of CHT and RT on the skeletal system as well as treatment-induced endocrine deficits (e.g., GHD, hypogonadism), reduced physical activity and altered muscle strength [7]. The use of GCS and nephrotoxic agents may cause hypercalciuria, which increases the risk of osteopenia and fractures. The prevalence of osteopenia increases especially in patients with ALL, CNS tumors and those undergoing hematopoietic stem cell transplantation (HSCT). Most studies on bone metabolism focus on children with ALL. These patients already show osteopenia at diagnosis, and this condition worsens with the beginning of treatment [10]. It is important to recognize and promptly treat hormonal alterations underlying bone metabolism, vitamin D deficiencies, and low calcium intake. Additionally, it is important to promote the beneficial effects of physical activity on bone remodeling.

## 6. Targeting Undernutrition

NS in pediatric cancer patients is challenging. Patients at high risk of undernutrition should be monitored closely [15]. If a patient is adequately nourished, is not losing weight, and is consuming at least 50% of the recommended nutritional intake, nutritional counselling by an expert dietitian is considered sufficient [28]. Nutritional counselling is also necessary for overweight and obese patients at diagnosis or during treatment, with special attention for patients taking long courses of steroids, who are at risk of sarcopenic obesity (ALL patients). Nutritional support, starting with oral supplements, is indicated when the patient does not have high-risk features, and is not able to meet 50% of the daily requirements orally [28]. However, this guideline is not followed by all centers. For example, commercial supplements, such as liquids, semi-solids or powders, energy-dense foods, or RUTFs could be used [15].

The appropriate way to nourish children with cancer is through the GI tract, which also has the important benefit of maintaining intestinal function and mucosal gut integrity [15]. Oral feeding cannot always be used in patients undergoing CHT or HSCT, due to the side effects of the therapies, such as nausea, vomiting and mucositis [29]. If oral feeding is not possible, alternative routes of nutrition and the associated risks should be discussed with parents and patients. The European Society for Clinical Nutrition and Metabolism (ESPEN) guidelines recommend enteral nutrition (EN) as a first choice if oral nutrition is inadequate and as long as the GI tract is not severely compromised, and this should be preferred over parenteral nutrition (PN) [62]. EN is now considered a safe and effective method to treat or prevent undernutrition [15]. The primary indications for EN are severely wasted or undernourished patients; children meeting less than 50% of their estimated nutritional requirements orally for more than five consecutive days; patients with over 5% weight loss since diagnosis; a decrease of >10% in the MUAC since diagnosis; or crossing of two growth percentiles over the course of treatment [28]. The choice of the type of EN must be based on individual needs and clinical characteristics: the type and size of the tubes, methods of enteral feeding (bolus vs. continuous), and type of formula (standard polymeric, concentrated, semi-elemental or elemental). There are several routes used for EN: nasogastric, nasoduodenal, and nasojejunal. The nasogastric tube represents the first choice that should be used for EN. Nasoduodenal and nasojejunal tubes should be utilized in patients at risk of pulmonary ingestion or with persistent vomiting. If a prolonged support is required (>4–6 weeks) or the nasopharynx needs to be bypassed, gastrostomy can be proposed [28]. EN (by tube, percutaneous endoscopic gastrostomy, or percutaneous endoscopic jejunostomy) can be provided by bolus feeding, continuous feeding, or a combination of these two methods. The bolus-feeding technique appears to be more physiological, as it mimics the oral route; however, continuous feeding is safer and better tolerated by patients [28].

Nutritional formula should be chosen based on the patient’s age and GI function [28]. In case of a functioning GI tract, standard polymeric formulas are the appropriate choice, as they contain intact proteins and long-chain triglycerides. On the other hand, in conditions of malabsorption, formulas containing amino acids and medium-chain triglycerides may be indicated. If fluid restriction is needed, or in case of reduced gastric capacity, concentrated formulas can be adopted; however, they are burdened with side effects caused by their osmolarity [28].

**Figure 3:**
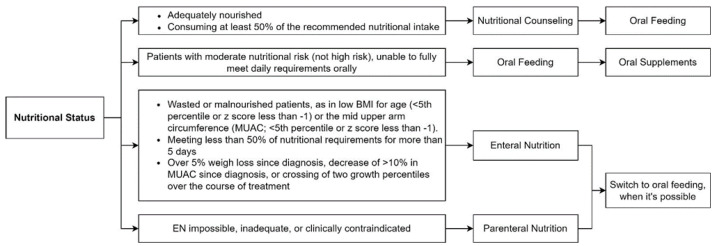
Possible nutritional interventions based on nutritional status.

EN is not always possible, such as in pediatric patients with GI obstruction or paralytic ileus, intractable vomiting or diarrhea, severe mucositis, intestinal GVHD, acute hemorrhage, severe pancreatitis, radiation enteritis, or GI perforation [15,62]. PN should be considered if EN is impossible, inadequate, or clinically contraindicated [15]. A retrospective study by Bendelsmith et al. investigated the use of EN in pediatric patients with newly diagnosed high-grade CNS tumors, medulloblastoma, or primitive neuro-ectodermal tumors (PNETs) [63]. The study highlighted that proactive enteral tube feeding led to a substantial increase in weight over the first year of treatment. Conversely, children who received rescue enteral tube feeding secondary to weight loss suffered a weight reduction and then regained it after the start of EN. Those who had not started EN even after losing weight had not regained the weight loss by the end of the follow-up. As a result, this study confirms the benefit of proactive enteral tube feeding [57]. In Figure 3, a scheme of possible nutritional interventions based on NS is provided.

The possible complications related to the use of PN are mechanical or equipment-related complications, such as central venous catheter (CVC) thrombosis, break, occlusion, or dislodgement; infective complications, like CVC-associated infections; and metabolic complications, for example deficiency or excess of PN components (hypertriglyceridemia and hyperglycemia), acid–base or electrolyte imbalance, drug interaction or compatibility problems, intestinal-failure-associated liver disease, and refeeding syndrome. PN can cause gut microbiome (GM) dysbiosis when used exclusively [15,28]. Additionally, PN can cause villous atrophy and increased intestinal mucosal permeability, leading to more infectious complications [21].

Several studies have compared EN and PN in pediatric cancer patients [21,58]. A multicenter prospective observational study and international survey investigated nutritional support in pediatric cancer patients with CHT-induced GI mucositis [21]. The study highlighted that there is variability in feeding strategies between hospitals, but, in most cases, EN was used as the first choice and PN was used only if tube feeding was not tolerated, such as in patients with vomiting. The results of the study showed that total PN led to better NS after CHT courses, compared to weight loss when no nutritional support or tube feeding only was given [21]. However, the use of total PN was correlated with more episodes of fever for which antibiotic treatment was started. Moreover, patients receiving PN were hospitalized for longer than those who did not receive total PN, but these patients were seen to have more severe mucositis, and were more severely ill, so this may have contributed to the prolonged hospitalization [21]. A study conducted by Zama et al. on a group of pediatric cancer patient allo-HSCT recipients compared the administration of EN and PN [58]. Oral feeding in the immediate post-transplant period cannot be used due to conditioning regimen-side effects, such as vomiting, anorexia, diarrhea, and mucositis. This work compared the post-transplant and nutritional outcomes of patients receiving EN for more than 7 days with patients receiving EN for fewer than 7 days or receiving only PN. Patients receiving EN showed a lower incidence of bloodstream infection (BSI) than those receiving PN in multivariate analysis. The principal sources of infections in pediatric cancer patients and HSCT recipients are CVC-related infections and the translocation of bacteria from endogenous intestinal flora to the bloodstream. The reduction of PN use may reduce CVC-related infections, thanks to the lower frequency in CVC handling. Furthermore, PN may induce gut mucosal atrophy and major dysbiosis, promoting bacterial translocation across the intestinal mucosal barrier and increasing the risk of intestinal bacterial domination of pathogens responsible for BSI. The protective role of EN against BSI could be explained by the eubiotic effect that EN has on the gut microbiome, stimulating the homeostasis of the intestinal ecosystem. Additionally, in patients receiving EN compared to those receiving PN, a shorter duration of nutritional support was observed, although it was not statistically significant. This resulted in early oral re-feeding and a greater reduction in BMI and BMI-z score after oral re-alimentation [64]. A recently published meta-analysis compared EN and PN after HSCT in pediatric and adult patients and shows a reduced incidence of acute graft-versus-host Disease (aGvHD), aGVHD grade III-IV, and gut aGVHD in patients receiving EN [65]. The protective role of EN against GVHD may be mediated by improved mucosal barrier integrity and increased intestinal eubiosis.

## 7. Nutrition and Gut Microbiome

The proper function of the GM is based on the equilibrium of many microbial species that adopt mutualistic strategies, known as eubiosis [66]. Eubiosis is characterized by a prevalence of beneficial species and a low prevalence of potentially pathogenic species. The composition of the GM is influenced by various factors, such as genotype, environment, and the diet of the individual. The microbiota not only directly influences numerous intestinal functions, but also acts remotely through its components and production of metabolites [66]. A reduction in non-pathogenic commensal species and resident bacterial species leads to the development of new bacterial populations and results in a dysregulation in the microbiome, known as dysbiosis [67]. Alterations in the function and composition of the gut microbiome may play a key role in the development of various diseases, including tumors. It is still unclear whether intestinal dysbiosis is a cause or consequence of cancer. The normal GM in children with cancer is disrupted, showing a reduction in healthy bacteria and a lower diversity both before and during treatment [68]. There is a suggested relationship between dysbiosis and carcinogenesis, in which alterations in the function of the immune system caused by dysbiosis can modify cell growth and the death process [68].

Alterations of the delicate GM balance can lead to pathologies such as the development of ALL in genetically predisposed subjects [69]. Intensive CHT associated with prophylactic or therapeutic antibiotic use plays a crucial role in inducing dysbiosis of the GM [70]. Recent studies suggest that gut microbiota dysbiosis may contribute to the GI toxicity following cancer treatments by causing an increase in proinflammatory cytokines (e.g., endotoxin) and immune cells that damage the GI tract, leading to a reduction in the barrier function of the intestinal epithelium and consequently an increased risk of bacterial translocation into the bloodstream [68].

Different types of nutrition can influence the impact on the microbiota and, therefore, the impact on cancer patients. A relevant distinction must be made between oral nutrition and artificial nutrition. Oral nutrition with increased energy and protein intake should be preferred in patients who are able to feed orally and are malnourished or at risk of undernutrition [71]. One of the most studied and practiced diets in our latitudes is the Mediterranean diet [72]. This is mainly characterized by the intake of fruit, vegetables, cereals and unsaturated fatty acids and a reduced intake of meat and saturated fatty acids. This type of nutrition decreases inflammatory signaling, and a large intake of carbohydrates produces short-chain fatty acids (SCFA) in the microbiota that positively affect cancer prevention and treatment [72]. These bacterial products act both intracellularly and extracellularly. At the intracellular level, for example, they act as direct inhibitors of histone deacetylases, promoting the differentiation of T-cells into cells producing IL-17, IFN-γ and IL-10. SCFAs, especially butyrate, have a direct effect on the intestinal mucosa, as they act as energy for the intestinal mucosal cells [73].

It has been observed that omega-3 polyunsaturated fatty acids, found in marine fish, vegetable oils, and seeds, have a beneficial role in maintaining gut health and modifying GM [74]. The benefits of omega-3 polyunsaturated fatty acids in cancer treatment have been well documented in multiple studies. These fatty acids have anti-inflammatory properties and have been shown to inhibit carcinogenesis and tumor growth. They play a vital role in cell membrane structure and are involved in important signaling processes and cell-to-cell interactions. In fact, these fatty acids are important components of enterocyte cell membranes and can modify the function of the intestinal mucosal barrier. Omega-3 polyunsaturated fatty acid supplementation has been linked to beneficial anti-inflammatory and antioxidant effects, which can help balance the immune system by influencing the intestinal microbes [74]. This is particularly relevant for patients undergoing chemotherapy or radiotherapy, as it may reduce the risk of gastrointestinal side effects caused by microbial dysbiosis.

An interesting study evaluated the impact of EN versus PN on GM composition in 20 pediatric patients undergoing HSCT, of whom 10 received EN and 10 received total PN [75]. It was found that patients who received EN after the transplant presented not only a faster recovery of their GM, but also the restoration of taxa that promoted health, such as *Faecalibacterium*, *Dorea*, *Blautia*, *Bacteroides*, *Parabacteroides*, and *Oscillospira* [75].

Severe neutropenia is a significant risk factor for infections in pediatric cancer patients who have undergone intensive CHT regimens [76]. Patients with dysbiosis may more easily experience neutropenic infections, such as neutropenic colitis. To reduce the risk of these infections in neutropenic patients, a low-bacterial-impact diet known as the neutropenic diet was developed [76]. Despite the premises for the development and application of the neutropenic diet, further studies have shown that the neutropenic diet does not provide any benefits and has no lower bacterial load than a normal diet [77,78]. Other studies have suggested that further investigations are needed to determine if it is an appropriate dietary choice, due to the lack of standardized international guidelines regarding nutrition in pediatric cancer patients [79,80].

The composition of the microbiome seems to influence patients’ survival from HSCT and its related complications (such as GVHD, infections, immune reconstitution, hepatic sinusoidal obstruction syndrome, febrile neutropenia, pulmonary complications, and relapse of the primary disease) [81]. Many GM-derived metabolites may play a key role in mediating important biological processes during HSCT. In particular, SCFAs can protect the intestinal epithelium and stimulate mucosal immunity. Both non-nutritional (such as antibiotics) and nutritional interventions can preserve GM from damage that may occur during HSCT or restore GM homeostasis after it [81].

Some recent studies have shown a relationship between dysbiosis and GVHD, noting a decline in bacterial species such as *Faecalibacterium* (belonging to the order of Clostridiales) with an increase in *Enterococcus* in patients who had GVHD. Enterococci, which can induce the production of IL-1 and IL-6, can lead to increased intestinal inflammation, while bacteria of the *Clostridiales* family have anti-inflammatory activity in the intestine, reducing the lethality of GVHD [82].

Treatments leading up to the HSCT procedure can substantially modify the GM of patients. These changes can lead to a state of dysbiosis with a decrease in *Faecalibacterium* and *Ruminococcus* in favor of bacteria such as *Enterococcus*, *Staphylococcus* and *Enterobacter*. There are also modifications involving molecules derived from bacteria such as SCFAs [69].

Some foods, such as ready-to-use therapeutic foods (RUTFs) can modulate the GM and restore eubiosis [10,70,83]. RUTFs have a significant role in correcting an unbalanced microbiota, improving the NS of the patients, and it has been identified that these can guarantee greater weight gain, improve body composition, and decrease episodes of FN, but data on the effect of RUTFs on GM are limited, and more research is needed in this area [10,70,83]. An interesting study was performed by Prasad et al. on children diagnosed with ALL, AML, Hodgkin and non-Hodgkin lymphoma, bone tumors, germ cell tumors and Wilms tumors [84]. One study arm was fed with standard nutritional therapy (SNT), while in the other study arm, in addition to the SNT, RUTFs were added. Moreover, in this case there was a greater weight gain in the SNT + RUTFs arm, as well as a decrease in infections, grade 3 and 4 mucositis, and treatment delays compared to SNT [84]. The RUTFs most used were milk powder, sugar, peanut butter, vegetable oil, vitamins, and minerals, which met the WHO recommendations for RUTF composition, according to a standard, energy-rich structure (ingredients are very dependent on local availability, cost and acceptability) [85].

From a dietary and nutritional perspective, it was possible to see how even the use of probiotics [68], such as *Bifidobacterium breve* strain (BBG-01), can influence the prophylaxis of infections in cancer patients, by decreasing the risk of infections in neutropenic patients and modulating the intestinal microflora by eradicating pathogenic microbial agents such as *Campylobacter*, *Candida*, and *Enterococcus* after oral administration [69].

However, studies on probiotics have limitations, and further research is needed to determine their real efficacy in modulating the GM of cancer patients [86]. Additionally, more safety data is needed regarding probiotics, especially due to the lack of knowledge about the risk of infections in giving live microorganisms to immunocompromised children and, therefore, the administration of probiotics should be done with great caution [69]. Nutritional supplementation of prebiotics, such as enteral formulas containing fructo-oligosaccharides (FOS), can provide a significant lactogenic effect and an increase in *Bifidobacteria.* This is believed to be due to an immunomodulation mechanism that stimulates the synthesis of IgA, increases the production of mucins, and modulates inflammatory cytokines, which leads to a decrease in the inflammatory state and positive physical growth [87].

Furthermore, SCFAs—GM-derived metabolites—play a key role in mediating important biological processes during HSCT. They can protect the intestinal epithelium from direct damage during HSCT and modulate mucosal immunity by decreasing alloreactivity (protection from acute and chronic GVHD) [81].

Table 6 summarizes the main nutritional intervention studies in pediatric patients with cancer.

Considering the overall available data, there is still limited knowledge about the role of gut dysbiosis in children with cancer and the effect of target therapies. More extensive studies are needed that take into account factors such as tumor type, tumor stage, treatment used, and environmental and genetic factors. Future research should also focus on the use of prebiotics and probiotics to improve gut dysbiosis in cancer patients, determining the optimal composition, dosage, and duration of treatment.

## 8. Conclusions

The alteration of NS is a modifiable risk factor that can be addressed with appropriate and timely intervention to improve the quality of life (QoL), immunological status, treatment response, and survival of children with cancer. Assessing NS is becoming an essential aspect of cancer care therapy, despite often taking a backseat to other oncological treatments and evaluations. Alterations in NS can have adverse effects not only at diagnosis, but also during survivorship [1]. Routine assessments during and after cancer treatment are necessary to ensure normal growth and development and measure the effective impacts on outcomes.

The goal of nutritional follow-up should be to identify individuals at an early stage who are at increased risk of malnutrition, considering their baseline nutritional evaluation, underlying pathology, and the necessary treatment [7]. Nutritional strategies are necessary for all children with a diagnosis of cancer, regardless of their initial body weight. Education of families and healthcare professionals on nutrition in children with cancer, as well as the adoption of tools and algorithms for nutrition interventions, should be promoted [10]. Early screening of NS should be a priority for all interdisciplinary oncological teams [1]. A close collaboration and sharing of expertise between pediatric oncologists and nutrition specialists, combined with careful and participatory sharing of the feeding experience with the family and the child (after age 6 years), is strongly required.

In regard to the microbiome, it is clear that maintaining eubiosis can limit bacterial translocations, infectious consequences, and immunological alterations, ultimately helping to maintain a better physical–nutritional state for patients undergoing oncological therapies [66]. This can be achieved through the use of foods such as RUTFs or probiotics [10,68].

Future studies should further clarify the pathophysiological mechanisms underlying malnutrition and changes in body composition during cancer treatment for different types and stages of cancer. A tailored nutrition plan that takes individual dietary variability into account would be a breakthrough in the therapeutic management of these patients. This could be achieved with the help of nutritional genomics, proteomics, and metabolomics. Currently, most nutritional recommendations are based on population data, but it is important to consider individual variability in dietary responses. Innovative technology could allow for the understanding of nutrient interactions with genes, proteins, and metabolites, leading to the formulation of individualized diets specific to the individual’s needs [7].

## Figures and Tables

**Table 1:** Definition of undernutrition in children (WHO).

Undernutrition	Acute undernutrition or wasting: WFH < −2 SD
	Chronic undernutrition or stunting: HFA < −2 SD
Overweight	<5 years: WFH > +2 SD
	5–19 years: BMI-for-age > +1 SD
Obesity	<5 years: WFH > +3 SD
	5–19 years: BMI-for-age > +2 SD

**Table 2:** Proposed timing of nutritional assessment during pediatric cancer treatment.

Treatment Required	Proposal
Intensive treatment or high risk of undernutrition	Every 3–4 weeks
Less intensive treatment	Every 3 months Every 6–12 months during maintenance
Children admitted to ICU	More frequent reassessments

Adapted from Fabozzi et al. [30]. ICU, intensive care unit.

**Table 3:** Proposed timing of nutritional assessment into survivorship [30].

Nutritional Risk	Proposal
No nutritional risks	First year: every 6 months After first year: annually
Presence of nutritional risk (i.e., inadequate eating habits, sedentary lifestyle, hypertriglyceridemia, high cholesterol levels) or well-nourished	First year: every 3 months Second to fifth year: every 6 months From fifth year onward: annually
Undernourished	Monthly assessment until normal nutrition status
Obese	Every 3 months

Adapted from Vianiet al. [32].

**Table 4:** Nutritional assessment in children with cancer: the A-B-C-D method.

Assessment		Advantages	Disadvantages
Anthropometric measures [8,16,33]	BMI, MUAC, TSFT, waist circumference, head circumference	Easily obtained	Measurement error and variability BMI may be altered in presence of oedema or of abdominal tumor mass
	BIA	Rapid, easy, inexpensive, no radiation exposure	No precision with oscillating hydration status and in chronically ill patients Standardization of execution required
	DXA	Accurate, less expensive than CT and MRI	Exposure to radiation albeit to a lesser extent than CT. It does not distinguish visceral from subcutaneous fat.
	CT	Accurate Lean body mass, subcutaneous fat, and visceral fat can be directly assessed	Exposure to radiation Expensive
	MRI	Accurate, no radiation exposure Lean body mass, subcutaneous fat, and visceral fat can be directly assessed	Expensive, longer image acquisition time
Biochemistry exams [7,8,29]	Liver and renal function test Lipid and glucose panel Serum protein concentration (prealbumin, albumin, RBP, transferrin) Micronutrients (B vitamins, vitamin D, calcium, zinc etc.)	Easily obtained (except for more specific laboratory exams, e.g., RBP dosage)	Influence of tumor itself or treatments More specific laboratory exams are not available in all centres
Clinical evaluation [31]	Muscle wasting Subcutaneous fat loss or excess Skin and hair changes Recent weight variation Presence of oedema Mucous membrane dryness Evidence of vitamin and mineral deficiencies Side-effect of cancer treatment affecting oral food intake	Easily obtained	Need for careful clinical examination and periodic re-evaluation
Dietary intake [36]	Intake of macro- and micro-nutrients Food aversions and preference Allergies or intolerances Current eating patterns Changes in physical activity level Family behavior and food hygiene at home	Allows dietary recommendations to be adapted to the patient’s needs and requirements	Need for expert personnel, such as dietitians or clinical nutritionists with expertise in this area, not available in all centers Need for periodic evaluation

BIA, bioelectrical impedance; BMI, body mass index; CT, computed tomography; DXA, dual-energy X-ray absorptiometry; MRI, magnetic resonance imaging; MUAC, middle-upper arm circumference; RBP, retinol-binding protein; TSFT, triceps skinfold thickness.

**Table 5:** Prevalence of undernutrition in different cancer types.

Type of Tumor	Average Prevalence of Undernutrition
Leukemia	5–10% Undernutrition ALL 7% Overnutrition ALL 2.9% Undernutrition AML 10.9% Overnutrition AML 14.9%
Neuroblastoma	20–50%
Solid tumors	34.3%
Brain tumors	31% Overnutrition 42.6% Obesity 40.4%
Other tumors	30%

ALL; acute lymphoblastic leukemia; AML, acute myeloid leukemia.

**Table 6:** Nutritional intervention studies in children with cancer.

References	Dietary Compound	Outcomes
Klement et al. [72]	Mediterranean diet	It decreases inflammatory signaling, producing SCFA that positively influences cancer prevention and treatment
Gupta et al. [77] Maia et al. [78]	Neutropenic diet	It does not bring benefits and does not contain a bacterial load lower than a normal diet
Barr et al. [53] Rajagopala et al. [70] Iddrisu et al. [83] Prasad et al. [84]	RUTFs	They can modulate the GM, restoring eubiosis, have a significant role in correcting an unbalanced microbiota, improving the NS of the patients, and can guarantee greater weight gain, improve body composition, and decrease episodes of FN
Bai et al. [68] Masetti et al. [69]	Probiotics	They can influence the prophylaxis of infections in cancer patients, but more safety data are needed regarding probiotics, especially due to the lack of knowledge about the risk of infections in giving live microorganisms to immunocompromised children
Zheng et al. [87]	FOS	They stimulate the synthesis of IgA, increasing the production of mucins, modulating inflammatory cytokines, which leads to a decrease in the inflammatory state and a positive physical growth

FN; febrile neutropenia; FOS, fructo-oligosaccharides; GM, gut microbiome; NS, nutritional status; RUTFs, ready-to-use therapeutic foods.

## Data Availability

Not applicable.
